# A new approach to econometric modeling in digitized consumer behavior

**DOI:** 10.3389/fpsyg.2022.940518

**Published:** 2022-11-29

**Authors:** Valentin Marian Antohi, Monica Laura Zlati, Romeo Victor Ionescu, Nicoleta Cristache

**Affiliations:** ^1^Department of Business Administration, Dunarea de Jos University of Galati, Galati, Romania; ^2^Department of Finance, Accounting and Economic Theory, Transylvania University of Brasov, Brasov, Romania

**Keywords:** online customer experience, econometric model, online purchase, cognitive states, affective states

## Abstract

Given how identifying motivational factors of online purchasing is critical to the success of online retailers, research on the antecedents of online customer experience (cognitive and affective experiential states) has attracted widespread attention. In this study, we conducted an extensive survey to identify major behavioral changes in the online buyer, and based on the age of the respondents we synthesized the findings into an econometric model to explain the impact of cultural, social, personal, and psychological traits on online purchasing. Our survey identified a myriad of motivational factors that influence online buyers' psychological perceptions and the impact of those factors has been reported. The proposed econometric model would help online retailers to better understand the motivational factors behind online customers' purchasing decisions. It also serves to inform the academic community of recent trends in this stream of research and shed light on future research.

## Introduction

There is a migration of the act of commerce from the classic retail area (specialized shops) to the online area, represented by e-commerce platforms. This is happening against the background of access to informational resources, especially on the Internet and networking sites. In this respect, the study, based on the questionnaire administrated to 199 people between April 2019 and April 2020, confirms the sensitive psychological dimension of the online buyer to several motivational factors. This study aims to identify the cognitive impact on the buyer induced by the motivational factors specific to the online environment, related to the psychological feedback of the visitor (potential buyer) on the products through the e-commerce platforms.

Some authors (Novak et al., 2000) define online customer experience as the “cognitive state experienced during navigation.” Other authors (Rose et al., 2012) suggest that it is a “psychological state manifested as a subjective response to the website.” Thus, online customer experience is a psychological state manifested as a subjective response to the web entrepreneur's value proposition (Gentile et al., 2007; Meyer and Schwager, 2007).

Some experts (Gentile et al., 2007) define the cognitive experiential state as a component of the online customer experience “connected with thinking or conscious mental processes” and the affective experiential state as the component of online customer experience that “involves one's affective system through the generation of moods, feelings, and emotions.”

On the other hand, a few authors such as Rose et al. (2012) identify the affective state of the experience with the state of mind, the feelings, and the emotions generated, so that the perceived advantages or value in use increase it positively.

Cognitive experiential states are reflected in this study as flow states. In this study the author (Csikszentmihalyi, 1990) defines the flow state as: “the state in which people are so intensely involved in an activity that nothing else seems to matter, the experience itself is so enjoyable that people will do it at great cost, for the sheer sake of doing it.”

A consumer in a state of flow has a feeling of happiness, confidence, and a desire for exploration, and also a distortion in the perception of time, which often occurs in the absence of time constraints when conducting a specific activity that provides positive feedback (Chou and Ting, 2003).

The state of flow is reflected in high levels of concentration, control, challenge, pleasure, and curiosity (Bridges and Florsheim, 2008; Hoffman and Novak, 2009). Consumers immersed in an online environment have strongly focused attention and the state of mind in which they find themselves is extremely rewarding. The state of flow is characterized by a distortion of time and the exclusion of daily concerns; the individual “temporarily loses self-awareness; his psychic energy is stimulated and diverted from what needs to be done”(Csikszentmihalyi and Csikszentmihalyi, 1988).

Evidence reveals that Internet users are primarily middle-aged and younger, even if their purchasing power is in many cases lower than older people. As a result, previous research has failed to highlight a significant age difference among online shoppers (Bellman et al., 1999; Li et al., 1999) or that online shoppers were older than traditional store shoppers (Donthu and Garcia, 1999; Bhatnagar and Ghose, 2004). Some authors (Zhou et al., 2007) empirically proved that the age difference between online and traditional consumers is diminishing, considering the effect of age on consumers' intention to purchase online, which remains to be further explored. Several research findings outlined a positive relationship between consumers' age and their likelihood to purchase products online (Stafford et al., 2004), while others revealed a negative relationship (Joines et al., 2003) or no relationship at all (Rohm and Swaminathan, 2004).

In terms of psychological patterns of consumer behavior affecting online customer sensitivity and impacting their acquisition decisions, the authors identified an increased research interest in adapting classical theories to the realities of a digitized society with a specific focus on global consumption (Table 1).

**Table 1:** Meta-analysis of the models from the relevant body of knowledge on the behavior of online consumers under the influence of motivational factors.

**Author**	**Year**	**Model**	**Impact**
Ng and Paladino, 2014	2009	The analysis is focused on electronic good consumption among students at an Australian university. These students were part from a marketing campaign, Mobile Muster and put into atention their concern about the environment. Finally, the analysis covers subjective norms, attitudes, perceived control, environmental concern, altruism, risk aversion, price consciousness, involvement, branding, environmental knowledge, and their relationship with purchase intentions.	The authors studied for the first time the eco-friendly achisition across the communication area. Moreover they pointed out the elements able to influence the consumers under an ecological approach. The study is very actual and has a good impact on the dedicated area of counsumption.
Darley et al., 2010	2010	The authors investigate online consumer behavior through decision-making processes (based on the influence of external factors) from a total of 52 published articles from 2001 to 2008	The impact of the research consisted of the magnitude of the study, but it is deficient in the interpretative part of the results in the sense of its focus on the realities of a continuously changing digitized economy.
Dennis et al., 2010	2010	The analysis is focused on the women as shoppers and their behavior. In this context, the women made their online shopping in an environment which is dominated by the male shoppers. On the other hand, the young females, will prefer social e-shopping to traditional e-shopping.	The study demonstrates the potential value of the concept of social e-shopping for future research. As a result, the impact of this approach is high.
Anaza, 2014	2014	The author analyzes the psychological processes to develop a predictive matrix of consumer behavior. The model carries out a vectorial mapping of the cognitive, affective and behavioral processes with the purpose of building the online consumer profile on psychological and behavioral perceptions	The impact of research is high through the vectorial approach of the model, its limits are found in the components that define consumer behavior and are included in the online acquisition management process.
Marc, 2015	2015	The author proposes a model based on integrated information systems–consumer behavior (IS-CB). This model is used for e-shopping and is able to examine the antecedents and consequences of e-shopping and usage behavior. The model takes into consideration a significant sample collected from e-shoppers.	The results of the model's implementation pointed out the importance of the entertainment gratification (EG), web irritation (WI), emotional state, and web atmospherics (WA) in the process of e-shopping. The impact is significant.
Stumpf and Baum, 2016	2016	The model proposed by the authors represents a novelty in the field as it combines the objective character of the brand evaluation with the subjective pecuniary issues of incentives and the psychological aspect of the trust gained in a previously evaluated brand. The three pillars of the model are combined on the principle of congruity in a reliable model and tested by pairs data analysis method	The impact is significant in this research area because the model combines factors from psychological perception with financial comfort factors and social stability factors (brand valuation), which gives a wide applicability to the model. The deficiency of the model consists in its unitary application on age groups, which constitutes a significant disturbing factor of the results.
Lemon and Verhoef, 2016	2016	The changing of the interaction between customer experience and the customer journey becomes esential for on-line entities, because the customer experiences are more social in nature. As a result, the companies have to change the business functions. The analysis is based on a very large literature review from a historical perspective within marketing.	The authors put together concepts as customer experience, customer journeys, and customer experience management in order to find and define critical areas for dedicated future research.
Gelbrich et al., 2017	2017	The authors conduct a research of motivation by financing the online consumer on retail chains	The impact is low considering the increasing access of consumers to the information and resistance they face to classical promotional methods, the specific weight of pecuniary factors being in decline in a digitized market (eg Black Friday).
Pham and Ahammad, 2017	2017	The authors approach the online consumer satisfaction model from a holistic perspective, analyzing downstream and upstream perceptual customer satisfaction following online purchases	The impact is high, generated by the research amplitude 7 hypotheses have been tested in several statistical hypostases. The findings reveal the impetus of consumer loyalty efforts that are de facto eroded both by transposing repetitive commerce and as a result of changing consumer behavior in relation to the diversified and ever-changing offer in the online environment. The authors consider that there are only two limitations of the study, namely the representativeness of the regional sample and the variability of the testing of the working hypotheses.
Jaiswal et al., 2018	2018	The authors analyze through a statistical model the perception of the consumer materialized in the confidence given to the seller and the products purchased, transposed through the modal procurement chain. The psychological perception of acquisition leads to increased loyalty if the modal chain components demonstrate viability.	The model has a significant impact, being demonstrated the e-loyalty mechanisms that can be exploited by marketers in building trust-building strategies. The limitations of the study are identified by researchers and consist of the regional specificity of the consumer profile (India), with some difficulty in extrapolating the study globally.
Lim et al., 2018	2018	The analysis in this paper is focused on the connections between the effects of message sequencing on attitudinal responses of the consumers. The whole analysis is based on a stratified random sample. The main result of the analysis is that the impact of the marketing messages in an emotional to rational sequence is the greatest. On the other hand, the corporate brand had no significant importance for the consumer attitudes.	The analysis in this paper brought as a new element the attitude of the consumers on genders. The impact of this approach is positive and able to support new dedicated analysis.
Bi, 2019	2019	The author has created an online consumer loyalty analysis model by analyzing the “black box” between the consumer's psychological perception based on the fundamental differences between different types of consumers and loyalty driven by consumer interests in relation to retailers in the online environment. The model is built on a neural network that analyzes the components of online consumer satisfaction emulate on their psychological perception with an impact on increasing consumer loyalty.	The impact of research is high through a statistically driven analysis, including hypothesis that leads to the idea that cumulation of community indicators can generate a traceability of retailers' loyalty in relation to their behavior regarding customer loyalty. The boundaries of the study consist of applying the questionnaire to a population in a semi-free market (China) that does not fully meet the conditions in other areas with other types of markets.
Ibrahim and Wang, 2019	2019	The authors have developed an analysis model based on time series of online consumer perception by accessing social networks (Twitter) along with the decoding of perceptions based on consumer choice	The model is innovative and impactful, considering the extent of social phenomena propagated through networking and marketing of the database. The model is stable by the quality of the information used and can be used successfully by marketers
Yang et al., 2019	2019	The authors have developed an impact model on the online consumer psychological perception based on quantification of utility and quality products in relation to their aesthetic functions	The model is descriptive, well argued, with pertinent conclusions of the predictive impact on the increase of loyalty according to the perceived utility of the products. The limitations of the study consist of psychological aspects, quantified by the financial leverage function, which can generate the subjective perception of the change in the consumer satisfaction curve on the global market.
Li et al., 2019	2019	The authors present a model of the motivational analysis of the redeeming factor applied to the online consumer based on the extrapolated model of the Markov chains.	The impact of the study is high and consists primarily in revitalizing some classical consumer behavioral theories considering the new challenges posed by relocating retailers' interest to the on-line environment. The limitations are
			presented in the study and aim at the regional applicability of the studied profile and the difficulty of generalizing at an extended level of research.
Jaiswal and Singh, 2020	2020	The authors analyze online retailing with the idea of identifying the major factors that affect the customers' satisfaction in India. For this purpose were used exploratory and multiple regressions methods.	The 7 assumptions made about customer satisfaction are logical, but too easy. The authors focus on identifying direct relationships and direct factors that influence satisfaction. We consider our approach a step forward in the dedicated comparative analysis of this study.
Becker and Jaakkola, 2020	2020	The authors conduct a meta-analysis covering 1,769 articles related to customer experience. The consolidation of the analysis has allowed hypotheses to be made which have enabled to establish a dual classification of research traditions that study customer experience as “responses to either managerial stimuli or consumption processes.”	The analysis suffers from being too theoretical. Unfortunately, the authors only make groupings of elements and hypotheses based on their study of the literature. The actual modeling of the phenomenon is missing, which we have overcome in our approach.
Becker and Jaakkola, 2020	2020	The concept of customer experience is analyzed using a meta-analysis of the literature. Based on the literature review, the authors realize a new conceptualization of customer experience connected to the consumer culture theory. Finally, the authors propose a conceptual model based.	As above, the authors present a limitation of the perspectives of the analysis due to the conceptual theoretical approach. Our present scientific approach performs the next step of the analysis, namely the quantification of the information and the implementation of an efficient model in the field.
Stein and Ramaseshan, 2020	2020	The effect of communication on customer experience is analyzed from doua perspective: utilitarian and hedonic motivation orientations.	We agree with the study's conclusion that technology offers a stronger hedonic motivation overall customer experience than utilitarian motivation.
Zhang and Chang, 2021	2020	An interesting connection between concepts such as globalization, new technologies, faster information and consumers' attitudes and behaviors is coherently realized. The analysis is supported by econometric and statistical tools.	The approach is interesting and we support it.
Liu et al., 2021	2021	The authors make the connection between customer experience, perceived value, and customer loyalty from a stimulus-organism-response (S–O–R) perspective in the case of Chinese consumers. The results of the analysis support the existence of a positive association between these three elements.	We consider the five hypotheses put forward by the authors to be appropriate and strongly connected to reality. We have taken a similar approach in the present scientific approach.
Bawack et al., 2021	2021	The authors analyse the connection between personality, trust, privacy concerns and prior experiences and how they affect customer experience. For this purpose, they use a contextualized theory of reasoned action (TRA-privacy), and propose a dedicated model which tested 224 US-based voice shoppers. The partial least squares analysis and structural equation modeling (PLS-SEM) and fuzzy-set qualitative comparative analysis (fsQCA) are the modeling methods used in this study.	We appreciate the authors' interesting mathematical approach for its complexity and accuracy.
Erdmann and Ponzoa, 2021	2021	Cost-benefit analysis applied in grocery ecommerce in connection to the Inbound Marketing actions is based on an application for optimal advertising budget from 1954. The authors adapt this approach to digital marketing and verified with empirical statistical analysis.	Even though the analysis covers several countries, the confirmation of the fitted model erroneously leads us to the idea that from 1954 to the present the modeling has not undergone obvious progress. We cannot support such an approach. As a result, our proposed model is new, viable, and efficient without being an update of another old model.
Zhao et al., 2021	2021	The authors consider it important to make the connection between consumers' conditions, factors, and behavioral reasons. In this respect, it uses the Adaptive Hybridized Intelligent Computational model	We appreciate the authors' approach, which we fully support. We also appreciate the complexity of the indicators analyzed.
		(AHICM), which models data relating to new products, new beliefs, and psychology for society.	
Janavi et al., 2021	2021	The impact of social media on online purchasing behavior is analyzed by the authors on the basis of 5 complex hypotheses. These hypotheses link personal consumer behavior to social media adoption, online purchase behavior, gender, age and medium type.	The authors' approach is classic and basically reiterates elements already known.
Molinillo et al., 2022	2022	Customer experience in connection to retailer mobile applications is a topic less addressed in the literature. The authors address this issue through the lens of cognitive, affective, relational and sensory analyses using PLS-SEM. The authors underline the novelty of the approach in the context of parameters as gender, age and device type.	We have reservations about PLS-SEM's ability to realistically model indicators as gender, age and device type in connection with customer experience.

The psychological evaluation models of consumer behavior discussed in the relevant literature provide insights into the various mechanisms of conditioning and loyalty of an online purchaser who is further influenced by subjective and objective factors that act together at a certain point resulting in online purchasing decisions. Against this background, this research aims to systematize the information presented in various specialized studies into a viable and easy-to-use tool that quantifies the impact of motivational factors on the behavior of the online consumer.

## Research sample

To meet the research objective of analyzing the psychological perception of the online buyer and examining the motivational factors influencing decision-making, a questionnaire was administered to 199 respondents from November 2019 to March 2020. Questionnaires were administered by e-mail to 250 participants, of which 199 sent valid feedback. The questionnaire was structured at three levels (level one—the buyer's choice in relation to the e-commerce offer; level two—the buyer's choice in relation to the motivational factors; and level three—demographic details including age and education, and annual average online shopping data).

The purpose of the study was to determine the age-specific consumer behavior transformed to online purchasing options which are influenced by a range of motivational factors. The goal was supported by the following research objectives:

*Objective 1*—To determine if the online buyer's psychological profile influences online purchase behavior.
*Objective 2*—To determine if there are differences in the online buyer's psychological profile based on age and education.
*Objective 3*—To identify the motivational factors influencing the online buyer's purchasing behavior.

**Figure 1:**
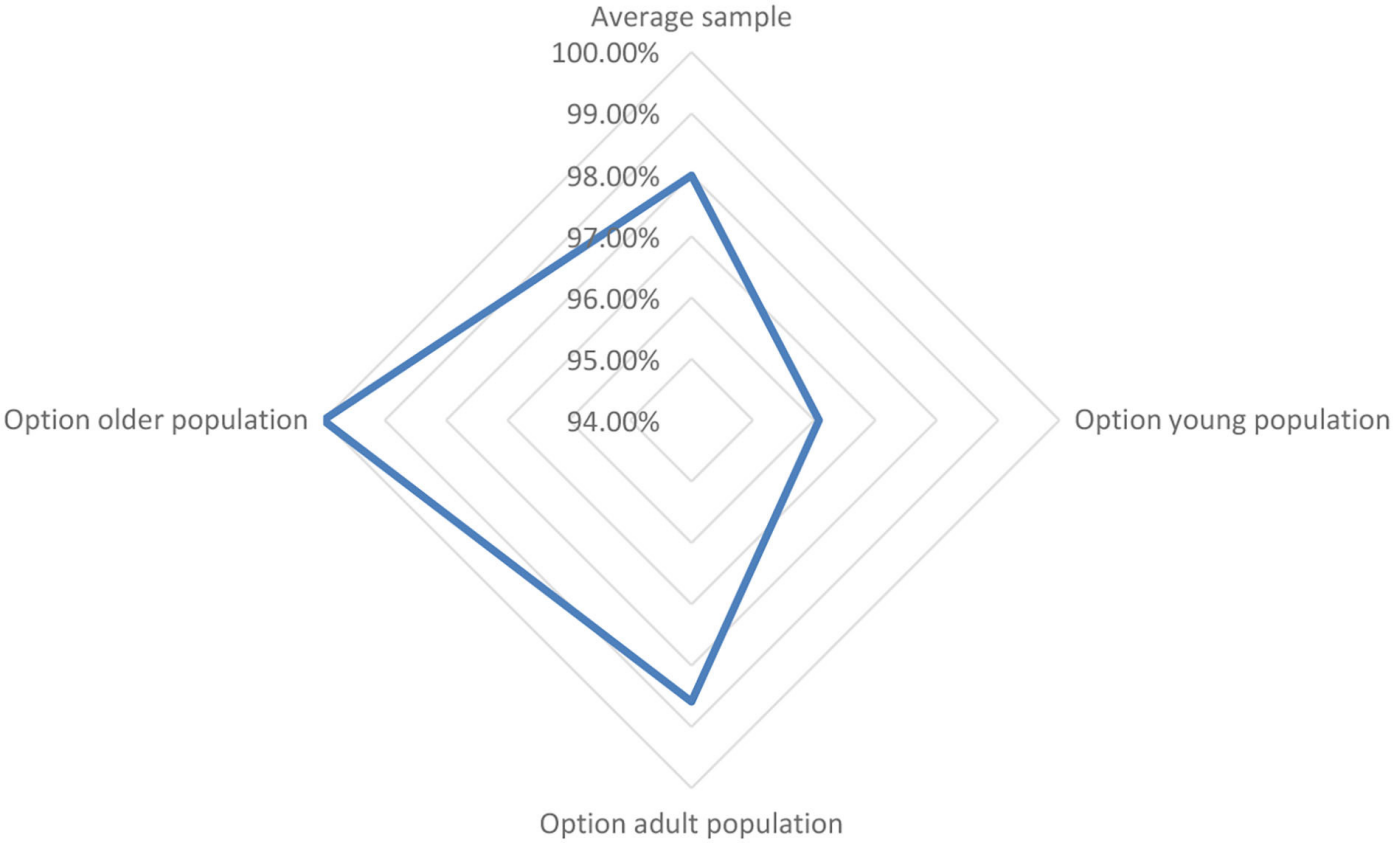
Online purchases related to respondents' age group.

The research sample was representative of the Romanian population. The minimum number of people who should have been questioned to ensure the 95% confidence rating range was 73. The determination of sample representativity was done using the Cochran WG formula: Romanian: population 19,000,000 people, expected incidence 95%, accuracy (A = 0.05), c = 1.96 for 95% confidence, minimum sampling population 72.99, rounded off to 73. Inclusion criteria: Romanian citizens, people who have Internet browsing habits on regular basis, and people using the online purchase environment. Exclusion criteria: people who are under the age of 18, people who did not complete primary education, and people who have never used the online purchase method (see Figure 1).

The study revealed the following structure based on the respondents' demography including age, education, sex, and access to online shopping. The vast majority (98%) of the respondents shopped online; younger respondents' online shopping was significantly lesser than that of older respondents.

## Research methodology

In order to achieve the objective of the study, namely identifying the cognitive impact on the buyer induced by the motivational factors specific to online shopping, related to the psychological feedback of the e-commerce platforms' visitors (potential buyers), we proposed an econometric model based on the following hypotheses:

1. there are no major differences in the online shopping behavior of buyers from different age categories (young, adult, and older adults).
2. all age categories manifest a similar amount of motivational factors regarding online acquisitions.
3. the cumulative cultural, social, personal, and psychological factors generate a unitary behavioral impact on the buyer.

The gaps in the psychological perception of online shopping in relation to motivational factors were analyzed for three age categories (young people, adults, and older adults) using an econometric model to determine the correlation between the dependent variable **v3** (psychological perception of adults, shopping in the online environment, in relation to motivational factors) and regression variables **v2** and **v4**. Where, **v2** was the psychological perception of the younger buyer operating in the online environment, in relation to the motivational factors and **v4** was the psychological perception of the older adult buyer operating in the online environment, in relation to the motivational factors.

We considered the control variable **v1** as the general perception of the whole sample, represented by the general averages for the whole population included in the research sample.

Each indicator in Table 2 was quantified for all respondents by age group by calculating the arithmetic averages, resulting in the variables of the model as follows.

**Table 2:** Research variables and their distribution within group age.

**Indicator acronym**	**Indicator**	**Numeric allocation of options**	**Perceptions of respondents**			**Average**
			**Under 25 years old**	**Between 25 and 55 years old**	**More than 55 years old**	
AO	Adherence for online acquisitions	Yes 1 No 2	1.04	1.01	1	1.02
FS	Flow state	Always 1 Never 7	3.47	3.52	3.6	3.51
HP	Happiness	Unhappy 1 Happy 7	5.75	5.73	5.83	5.74
PA	Passion	Melancholic 1 Passionate 7	5.24	5.27	5.2	5.26
GT	Gratitude	Annoyed 1 Contented 7	6.02	5.75	6	5.83
EN	Energy	Sluggish 1 Frenzied 7	5.14	5.32	5.6	5.28
PT	Patience	Calm 1 Excited 7	5.47	4.93	5.5	5.08
ST	Stimulation	Relaxed 1 Stimulated 7	4.3	4.48	5.33	4.46
AU	Autonomy	Guided 1 Autonomous 7	4.67	5.17	5.33	5.04
IN	Influence	Influenced 1 Influential 7	4.29	4.28	4.17	4.28
DO	Demand/offer	Totally disagree 1 Totally agree 7	6	5.77	5.5	5.82
DA	Desire to act	Totally disagree 1 Totally agree 7	5.76	5.62	5.83	5.66
RE	Resignation	Totally disagree 1 Totally agree 7	3.27	2.97	2.83	3.05
MCF	Motivation based on communication factors	Totally disagree 1 Totally agree 7	4.71	4.72	5.5	4.74
MVI	Motivation through relevant visual information	Totally disagree 1 Totally agree 7	5.14	5.33	5.8	5.29
MR	Minimal risk	Totally disagree 1 Totally agree 7	4.16	4.48	5.67	4.43
AP	Acceptance of promises	Totally disagree 1 Totally agree 7	4.08	4.52	5.5	4.43
PS	Perception of security	Totally disagree 1 Totally agree 7	4.25	4.98	6.17	4.83
CGOT	Confidence gained after online transactions	Totally disagree 1 Totally agree 7	4.22	4.94	6	4.79
GE	Global experience	Totally disagree 1 Totally agree 7	5.28	5.63	5.83	5.55
CE	Consumer education	Totally disagree 1 Totally agree 7	4.88	5.23	5.5	5.15
SRCP	Satisfaction regarding commercial procedures	Totally disagree 1 Totally agree 7	5.4	5.77	6	5.69
ASE	After-sales experience	Totally disagree 1 Totally agree 7	5.18	5.28	6.17	5.28
PROCA	The probability of repeating the online commerce act	Totally disagree 1 Totally agree 7	5.96	6.36	6.33	6.26
ARACO	Anticipating the repeat of the online commerce act	Totally disagree 1 Totally agree 7	5.98	6.33	6.34	6.24
CR	Customer retention	Totally disagree 1 Totally agree 7	5.29	5.83	6	5.7
TR	Temptation of repurchasing	Totally disagree 1 Totally agree 7	5.67	5.83	6.33	5.8
AGE	Age	Under 25 years old: 1 Between 25 and 34 years old: 2 Between 34 and 44 years old: 3 Between 44 and 54 years old: 4 More than 55 years old: 5	1	2.77	5	2.39
GN	Gender	Male 1—Female 2	1.75	1.65	1.67	1.67
STD	Studies	Primary studies Secondary studies Bachelor Postgraduate	2.96	2.98	3	2.97
FOA	Yearly frequency of online acquisitions	Between 1 and 6 on-line purchases per year: 1 Between 6 and 10 on-line purchases per year: 2 More than 10 online purchases per year: 3	2.08	2.37	2.33	2.29

The data were integrated into a regression model, based on the ordinary least squares (OLS) method:


(1) $$\begin{array}{l} \text{v3}=\;\alpha \;*\text{v2}+\;\beta \;*\text{v4}+\;\varepsilon \end{array}$$

- v3 (dependent variable): The psychological perception of adult buyers operating in the online environment in relation to motivational factors and regression variables v3 and v1.
- v2 (regression variable): Psychological perception of the younger buyer operating in the online environment, in relation to the motivational factors.
- v4 (regression variable): The psychological perception of the older adult buyer operating in the online environment, in relation to the motivational factors.
- α, β—the regression coefficients of the model.
- ε—the residual variable.

## Results

The data were modeled using the Gretl statistical program, version 3 adapted in 2018 for Windows 10, to obtain the regression equation defined as follows:


$$\begin{array}{l} \hat{\;}v3=+0.559*v2+0.431*v4\\ \;(0.0397)(0.0351)\\ n=32,R\text{-squared}=0.999\\ \left(\text{standard errors in parentheses}\right) \end{array}$$

According to the regression equation, the statistical representativeness of the model for 32 observations and 3 variables was 99.9%, which meant that the psychological perception of the buyer was homogeneous across all age categories, especially based on the similar perception of the motivational factors for purchases made in the online environment. The model was tested for statistical homogeneity and relevance tests, obtaining the data below:

**Model 1: OLS, using observations 1–32**

**Dependent variable: v3**

**Table:** 

	**Coefficient**	**Std. Error**	***t*** **-ratio**	***p*** **-value**	
v2	0.558815	0.0396733	14.0854	< 0.00001***
v4	0.430644	0.0351103	12.2655	< 0.00001***
Mean dependent var	4.619306	S.D. dependent var		1.375548	
Sum squared resid	1.085474	S.E. of regression		0.190217	
R-squared	0.998536	Adjusted R-squared		0.998487	
*F*_(2, 30)_	10231.29	*P*-value(F)		3.04e−43	
Log-likelihood	8.733479	Akaike criterion		−13.46696	
Schwarz criterion	−10.53549	Hannan-Quinn		−12.49526	

The values of *p* were < 0.00001 for both regressions, indicating a high degree of significance of the model in which the value of *R*^2^ was very close to the unit value (0.9985), and the standard deviation of the model-dependent variable was 1.3755.

The heteroscedasticity test revealed that in the hypothesis zero heteroflexibility was not present, which demonstrated that the model was representative of determining the psychological profile of the online buyer.

**Breusch-Pagan Test for Heteroscedasticity:**

Null hypothesis: heteroscedasticity not present

Statistic Test: LM = 2.61235

with *p*-value = *P* (Chi-square (1) > 2.61235) = 0.106035

The normally distributed residual variable test shows that in the null hypothesis the error was normally distributed, and the value of Chi-square (2) was 1.9 which demonstrated the homogeneity of the model and the residual variable minimal value.

**Test for normality of residual variable:**

Null hypothesis: error is normally distributed

Chi-square (2) test = 1.90395

with *p*-value = 0.385978

The 95% confidence interval forecast shows the homogeneity of the trend evolution as compared to the 95% confidence estimate, which demonstrated that the model was viable, and the results were tested for high significance using the predictive method. The *Q*–*Q* plot diagram represented an identifier of the linear trend in the case of the studied phenomenon, especially since the placement on the right of the trend does not generate large amplitude deviations.

The distribution frequency for the dependent variable indicated in the graph above for the 32 observations is presented in Table 3.

**Table 3:** Distribution frequency of the dependent variable.

**Interval**	**Midpt**	**Frequency**	**Rel**.	**Cum**.
< -0.43101	−0.49753	1	3.13%	3.13%
−0.43101 to −0.29796	−0.36448	0	0.00%	3.13%
−0.29796 to −0.16491	−0.23143	5	15.63%	18.75%
−0.16491 to −0.031857	−0.098382	7	21.88%	40.63%
−0.031857 to 0.10119	0.034668	9	28.13%	68.75%
0.10119 to 0.23424	0.16772	6	18.75%	87.50%
≥0.23424	0.30077	4	12.50%	100.00%

Frequency distribution for uhat1, obs 1–32.

Number of bins = 7, mean = 0.00293412, SD = 0.190193.

**Figure 2:**
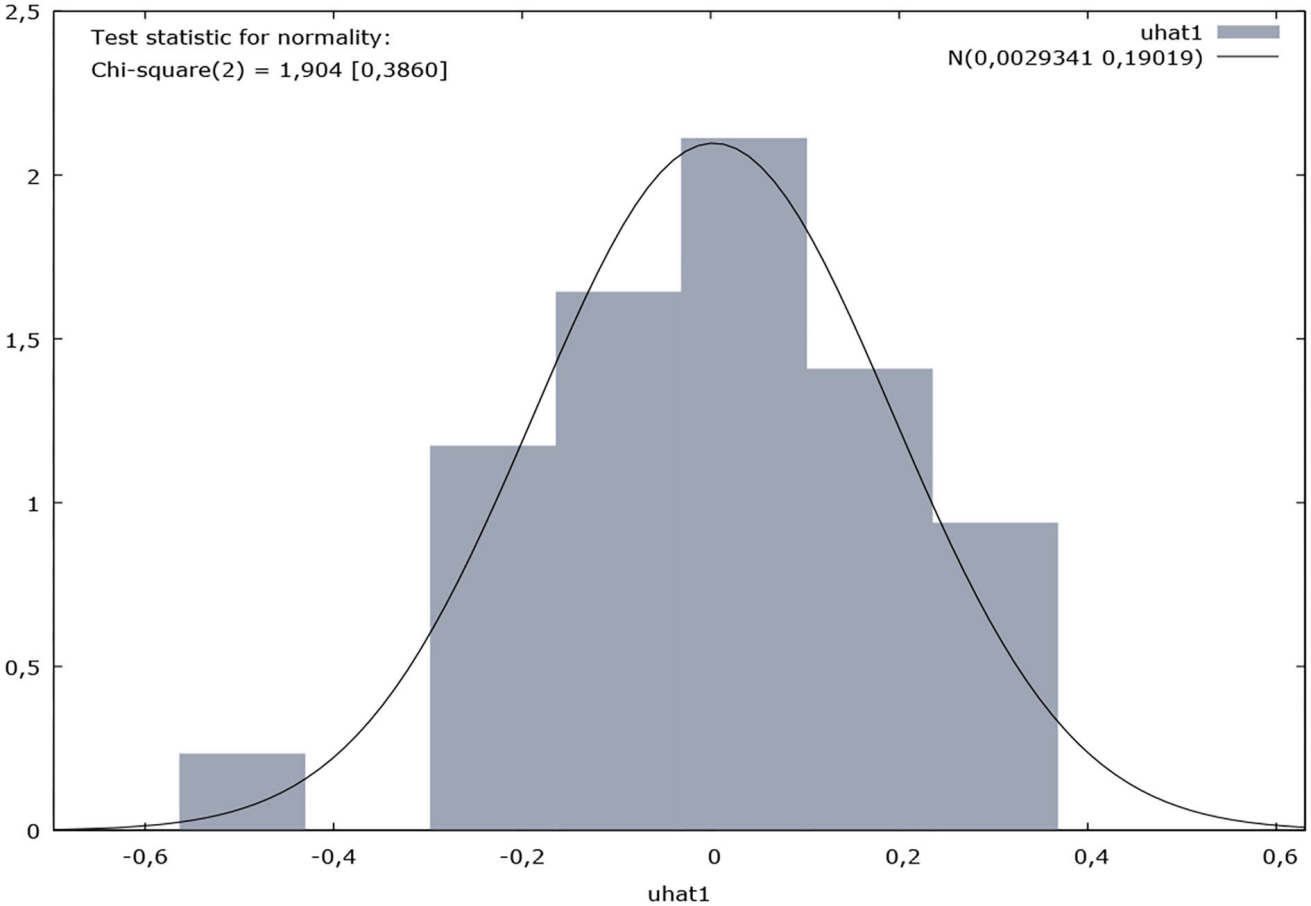
Histogram distribution.

The test for the null hypothesis in the normally distributed version showed a Chi-square value (2) of 1,904 with a *p*-value of 0.38598, resulting in a histogram distribution diagram of the frequencies, as follows in Figure 2.

The histogram distribution reflects the homogeneity of the model.

The model data was tested by the curve fit statistic option on the SPSS program. The results confirmed the hypotheses of the study, as follows. Based on the report of the application of the model on pairs of data using the linear curve function, linear logarithmic, and quadratic modeling, as well as by the *S*-test, the values of the degree of significance of the general model of perceptions concerning the individual differed on respondents' age intervals (18–25, 25–55, and 55–75 years). The values reflected a high degree of statistical significance, the average age range being the one that best suits the 99.5% overall profile, motivated by the financial support of the buy option. The second age bracket with a 95.1% statistical significance was the profile of 18–25 year-olds whose income is insured independently or by the family. The lowest degree of statistical significance was found in the older adult population, the model representativeness being in this case 83.00% (Table 4).

**Table 4:** Model summary (curve fit statistic): The independent variable is general perception.

	**R**	**R square**	**Adjusted R square**	**Std. error of the estimate**
Perceptions of respondents under 25 years old	0.975	0.951	0.949	0.324
Perceptions of respondents between 25 and 55 years old	0.998	0.995	0.995	0.096
Perceptions of respondents above 55 years old	0.911	0.830	0.825	0.605

The ANOVA test reflects the profile predictability in relation to the general profile. The adult population aged 25–55 was ranked first, followed by the young population aged 18–25, and the older adult population aged over 55.

**Figure 3:**
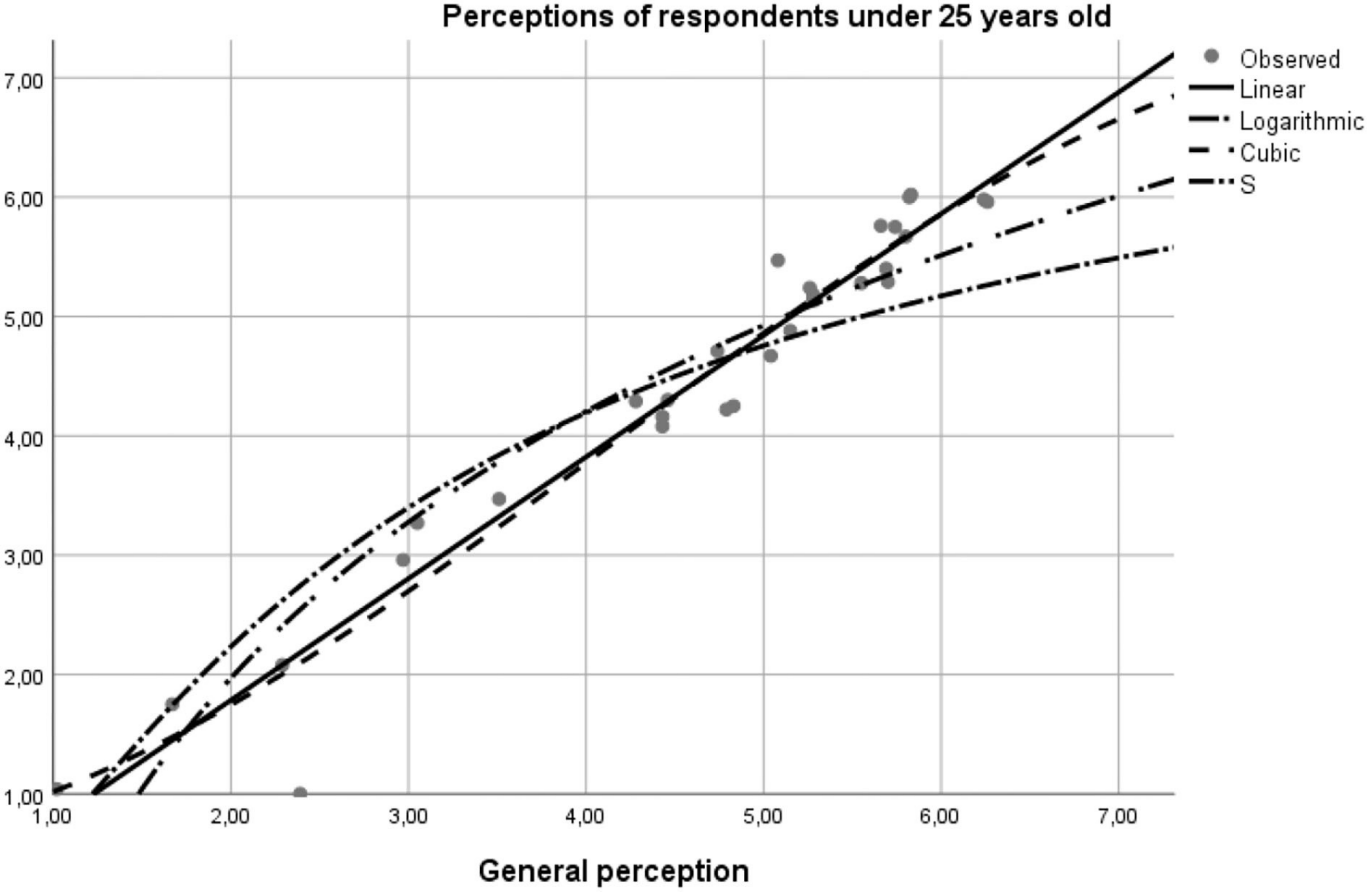
The curve fit diagram for the general model against the model regress for the population under 25 years of age.

**Figure 4:**
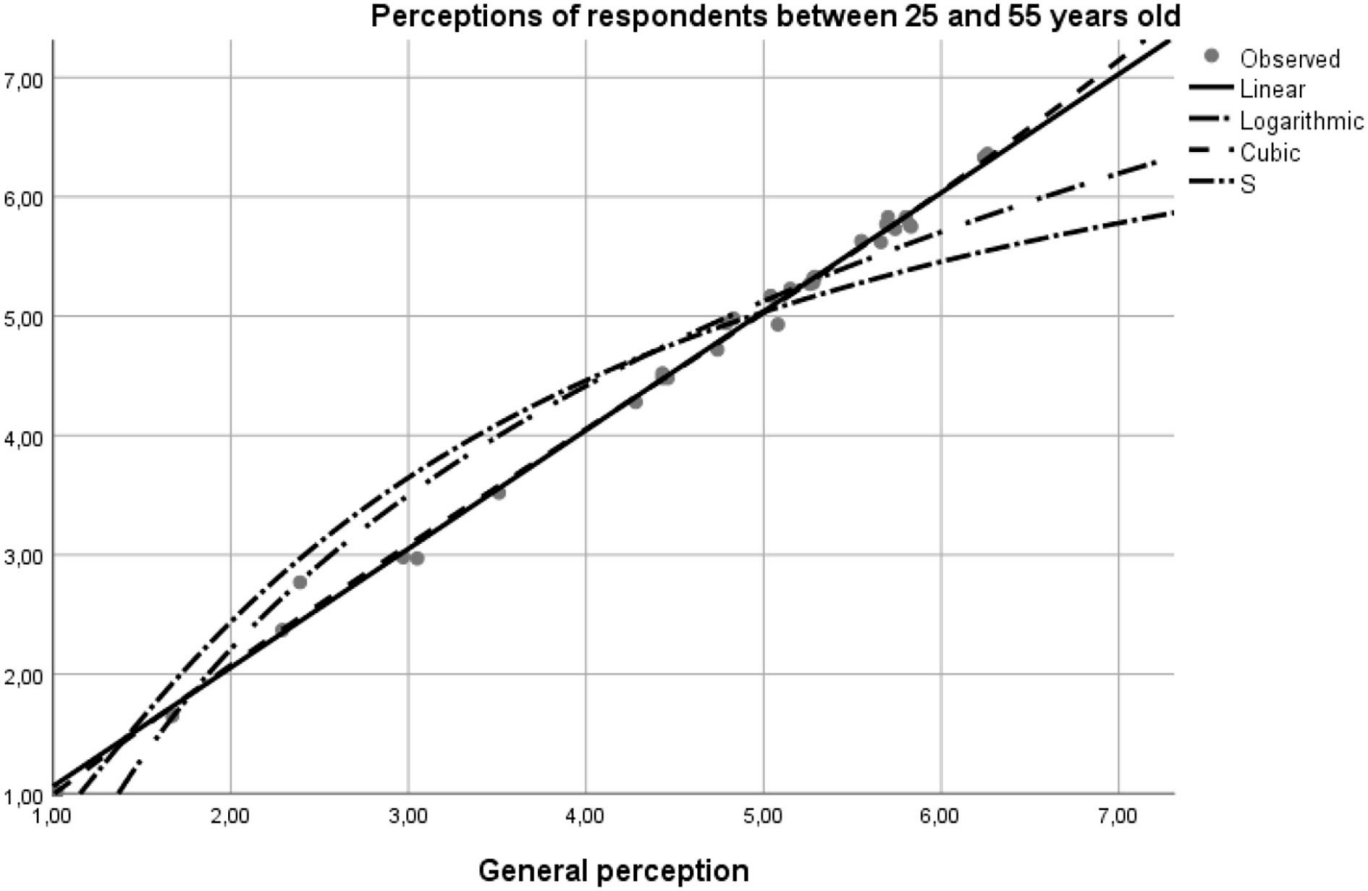
The curve fit diagram for the general model vs. the model regressor for the 25–55 year-old population.

**Figure 5:**
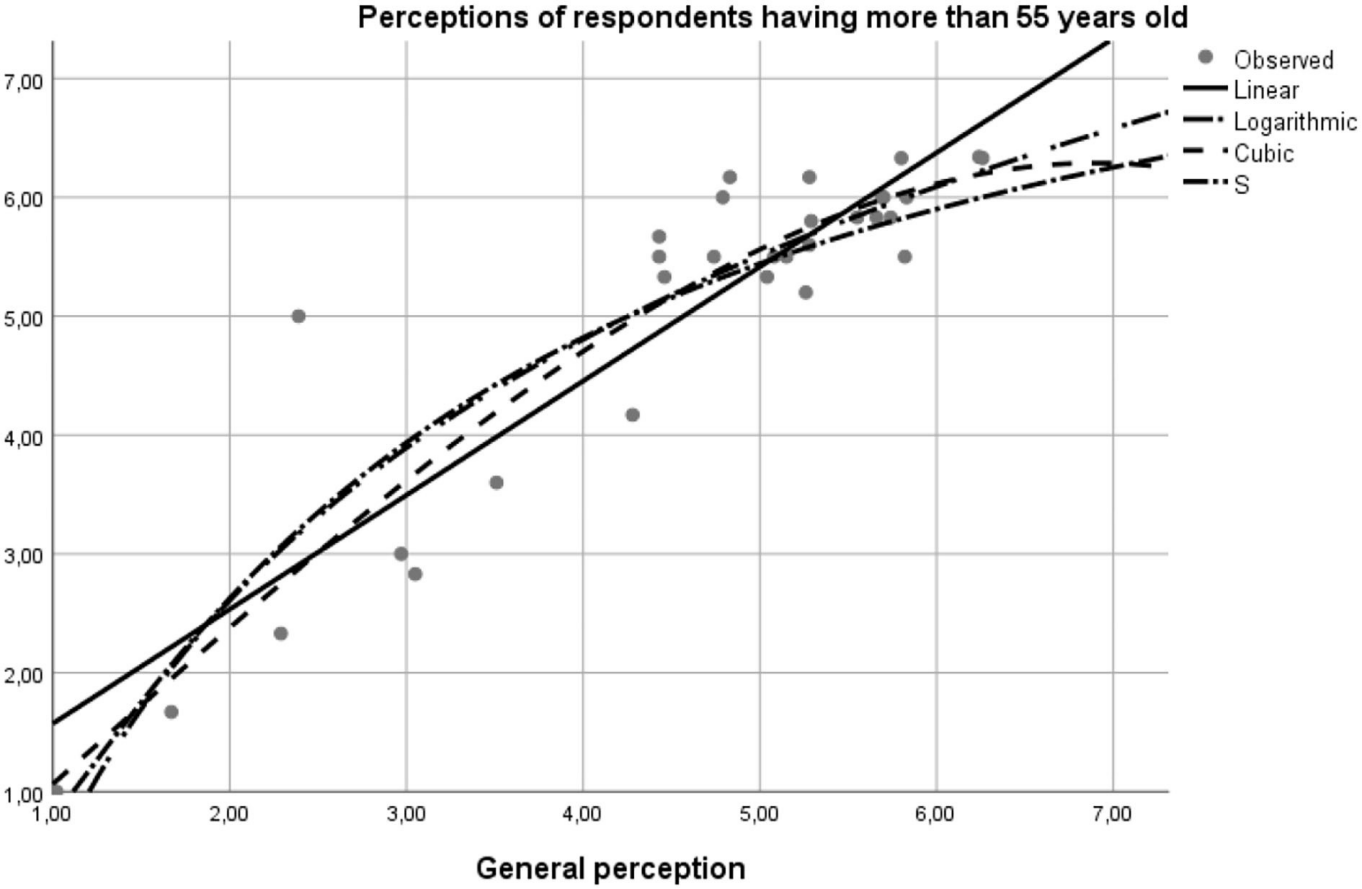
The curve fit diagram for the general model against the model regression for the population aged over 55.

Structured on age scales, the curve fit diagram generated the following results:

- **Perceptions of respondents under 25 years old—the matching with the general model is 97.5%, according to the linear Beta coefficient on the linear function with the value of the regression coefficient 1.019 and constant** –0.253 (*y* = 1.019^*^x **– 0.253)** (Figure 3).
- **Perceptions of respondents between 25 and 55 years old—matching the general model is 99.8%, according to the standardized Beta coefficient on linear function with the regression coefficient value of 0.995 and the constant +0.067 (*y* = 0.995** ^*^ ***x*** **+ 0.067)** (Figure 4).
- **Perceptions of respondents over 55 years of age—matching the general model is 91.1%, according to the Beta standardized linear function with the regression coefficient 0.961 and the constant of +0.613 (*y* = 0.961** ^*^ ***x*** **+ 0.613)** (Figure 5).

All the statistical tests carried out led to the conclusion that the general profile detached from the observation of the 199 questionnaires administered to the respondents was homogeneous, well determined by the models structured on age scales (spectral analysis), thus confirming the hypotheses of the model as follows:

1. In the presence of the motivational factors there is a lack of major discrepancies in the behavior of the buyer of online products according to the age of respondents, in the three major categories of age: young, adult, and older adults.
2. All age segments show a similar amplitude of motivational factors for online purchases.
3. The cumulative impact of cultural, social, personal, and psychological factors generates a unitary behavior in the buyer.

## Discussion

The study reflected, at the level of the structural indicators, the following relevant issues. First, the flow rate of online buyers was present in the sample population at a mean level of significance (represented by an average frequency of occurrence of the phenomenon). The maximum point of distribution on the 7-stage occurrence scale was in the middle of the interval (step 4), with symmetric left-hand distributions of relatively homogeneous frequencies. The scale-based approach is one generally used in the study of consumer behavior (Darley et al., 2010; Marc, 2015; Jaiswal et al., 2018), allowing the transformation of qualitative variables into quantitative variables, with the subsequent possibility of modeling outputs.

An overview of the motivational factors, harmonized in amplitude by factor classes, is presented in Table 5. Depending on the impact of the factors on the psychological profile, we categorized it as M1, M2, and M3, where M1 included motivational factors that had a strong psychological impact on the online buyer's behavior, M2 included motivational factors that had an average psychological impact on online buyer's behavior, and M3 included motivational factors that had a low psychological impact on online buyer's behavior.

**Table 5:** Histogram distribution of minimum–maximum perception patterns based on age vs. general model.

**Motivational factor range**	**Perceptions of respondents under 25 years old**	**Perceptions of respondents between 25 and 55 years old**	**Perceptions of respondents having more than 55 years old**	**Average**
M1	Adherence for online acquisitions (frequently yes)—minimum frequency	Adherence for online acquisitions (frequently yes)—average frequency	Adherence for online acquisitions (yes)—maximum frequency	Adherence for online acquisitions (frequently yes)—average frequency
M1	Flow state (sometime)—maximum frequency	Flow state (sometime)—average frequency	Flow state (sometime)—minimum frequency	Flow state (sometime)—average frequency
M1	Happiness (happy)—average frequency	Happiness (happy)—minimum frequency	Happiness (happy)—maximum frequency	Happiness (happy)—average frequency
M1	Passion (slightly passionate)—average frequency	Passion (slightly passionate)—maximum frequency	Passion (slightly passionate)–minimum frequency	Passion (slightly passionate)—average frequency
M1	Gratitude (contented)—maximum frequency	Gratitude (contented)—minimum frequency	Gratitude (contented)—average frequency	Gratitude (contented)—average frequency
M1	Energy (slightly frenzied)—minimum frequency	Energy (slightly frenzied)—average frequency	Energy (frenzied)—maximum frequency	Energy (slightly frenzied)—average frequency
M3	Patience (slightly excited)—average frequency	Patience (slightly excited)—minimum frequency	Patience (excited)—maximum frequency	Patience (slightly excited)—average frequency
M1	Stimulation (slightly stimulated)—minimum frequency	Stimulation (slightly stimulated)—average frequency	Stimulation (slightly stimulated)—maximum frequency	Stimulation (slightly stimulated)—average frequency
M2	Autonomy (slightly autonomous)—minimum frequency	Autonomy (slightly autonomous)—average frequency	Autonomy (slightly autonomous)—maximum frequency	Autonomy (slightly autonomous)—average frequency
M2	Influence (slightly influential)—maximum frequency	Influence (slightly influential)—average frequency	Influence (slightly influential)—minimum frequency	Influence (slightly influential)—average frequency
M1	Demand/offer (totally agree)—maximum frequency	Demand/offer (totally agree)—average frequency	Demand/offer (totally agree)—minimum frequency	Demand/offer (totally agree)—average frequency
M1	Desire to act (totally agree)—average frequency	Desire to act (totally agree)—minimum frequency	Desire to act (totally agree)—maximum frequency	Desire to act (totally agree)—average frequency
M3	Resignation (neutral)—maximum frequency	Resignation (neutral)—average frequency	Resignation (neutral)—minimum frequency	Resignation (neutral)—average frequency
M2	Motivation based on communication factors (medium agree)—minimum frequency	Motivation based on communication factors (medium agree)—average frequency	Motivation based on communication factors (totally agree)—maximum frequency	Motivation based on communication factors (medium agree)—average frequency
M2	Motivation through relevant visual information (medium agree)—minimum frequency	Motivation through relevant visual information (medium agree)—average frequency	Motivation through relevant visual information (totally agree)—maximum frequency	Motivation through relevant visual information (medium agree)—average frequency
M3	Minimal risk (medium agree)—minimum frequency	Minimal risk (medium agree)—average frequency	Minimal risk (totally agree)—maximum frequency	Minimal risk (medium agree)—average frequency
M2	Acceptance of promises (medium agree)—minimum frequency	Acceptance of promises (medium agree)—average frequency	Acceptance of promises (totally agree)—maximum frequency	Acceptance of promises (medium agree)—average frequency
M3	Perception of security (medium agree)—minimum frequency	Perception of security (medium agree)—average frequency	Perception of security (totally agree)—maximum frequency	Perception of security (medium agree)—average frequency
M1	Confidence gained after online transactions (medium agree)—minimum frequency	Confidence gained after online transactions (medium agree)—average frequency	Confidence gained after online transactions (totally agree)—maximum frequency	Confidence gained after online transactions (medium agree)—average frequency
M2	Global experience (medium agree)—minimum frequency	Global experience (totally agree)—average frequency	Global experience (totally agree)—maximum frequency	Global experience (totally agree)—average frequency
M2	Consumer education (medium agree)—minimum frequency	Consumer education (medium agree)—average frequency	Consumer education (totally agree)—maximum frequency	Consumer education (medium agree)—average frequency
M1	Satisfaction regarding commercial procedures (medium agree)—minimum frequency	Satisfaction regarding commercial procedures (totally agree)—average frequency	Satisfaction regarding commercial procedures (totally agree)—maximum frequency	Satisfaction regarding commercial procedures (totally agree)—average frequency
M2	After-sales experience (medium agree)—minimum frequency	After-sales experience (medium agree)—average frequency	After-sales experience (totally agree)—maximum frequency	After-sales experience (medium agree)—average frequency
M1	The probability of repeating the online commerce act (totally agree)—minimum frequency	The probability of repeating the online commerce act (totally agree)—maximum frequency	The probability of repeating the online commerce act (totally agree)—average frequency	The probability of repeating the online commerce act (totally agree)—average frequency
M2	Anticipating the repeat of the online commerce act (totally agree)—minimum frequency	Anticipating the repeat of the online commerce act (totally agree)—average frequency	Anticipating the repeat of the online commerce act (totally agree)—maximum frequency	Anticipating the repeat of the online commerce act (totally agree)—average frequency
	Customer retention (medium agree)—minimum frequency	Customer retention (totally agree)—average frequency	Customer retention (totally agree)—maximum frequency	Customer retention (totally agree)—average frequency
M1	Temptation of repurchasing (totally agree)—minimum frequency	Temptation of repurchasing (totally agree)—average frequency	Temptation of repurchasing (totally agree)—maximum frequency	Temptation of repurchasing (totally agree)—average frequency
	Age (under 25 years old)	Age (between 34 and 54 years old)	Age (more than 55 years old)	Age (between 34 and 54 years old)
	Gender (female)—maximum frequency	Gender (mixed preponderent female)—minimum frequency	Gender (mixed preponderent female)—average frequency	Gender (mixed preponderent female)—average frequency
	Studies (bachelor)—minimum frequency	Studies (bachelor)—average frequency	Studies (postgraduate)—maximum frequency	Studies (bachelor)—average frequency
M1	Yearly frequency of online acquisitions (between 6 and 10 on-line purchases per year)—minimum frequency	Yearly frequency of online acquisitions (between 6 and 10 on-line purchases per year)—maximum frequency	Yearly frequency of online acquisitions (between 6 and 10 on-line purchases per year)—average frequency	Yearly frequency of online acquisitions (between 6 and 10 on-line purchases per year)—average frequency

**Figure 6:**
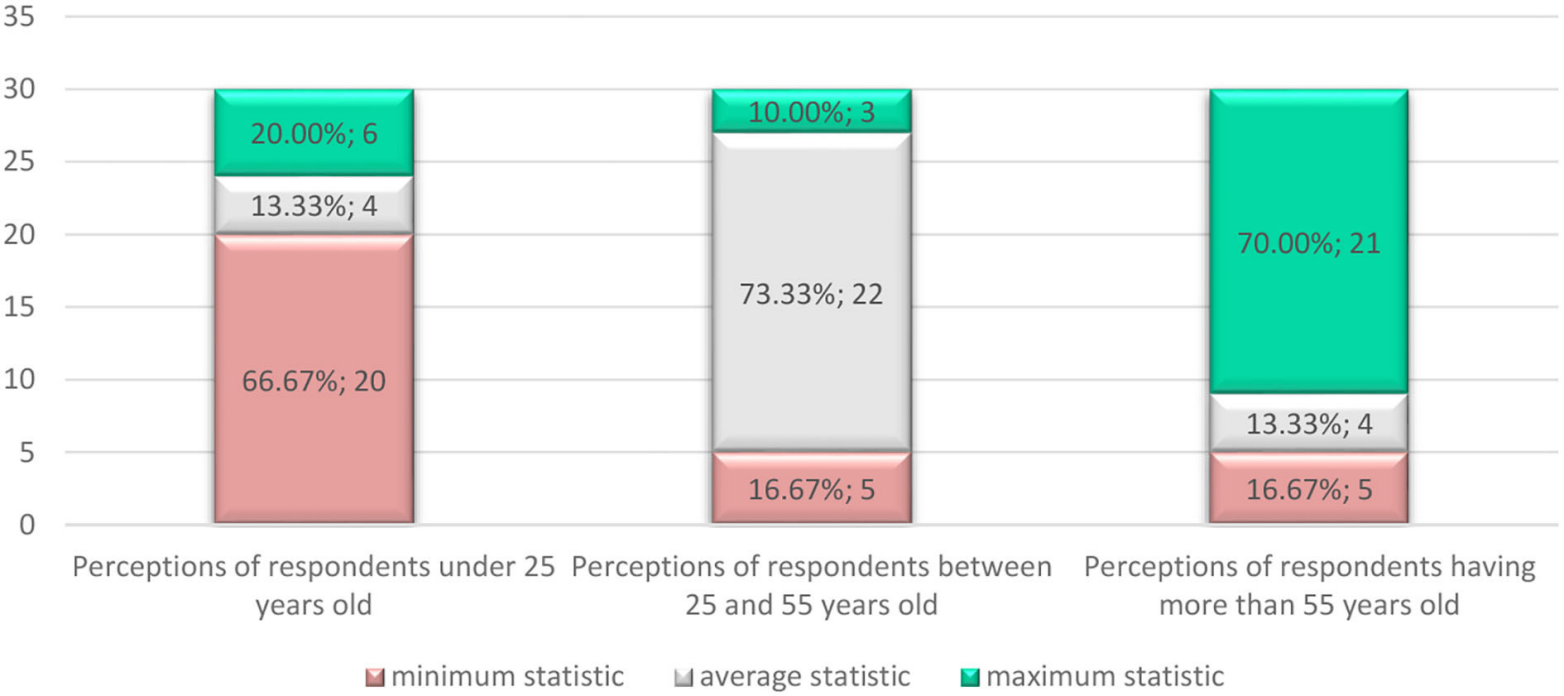
Representation of the general profile and segmented profiles according to the age criterion according to the minimum-maximum values obtained for 30 variables of the questionnaire from a total of 31, except for the age.

Second, customer satisfaction depended to a large extent on the quality of services offered by the seller. To be able to excel in sales, the seller must exceed buyers' expectations of the quality of the services and articles offered. This approach would lead to buyers returning for future shopping as well as engaging in word-of-mouth dissemination that the seller is trusted and attract new potential customers (Figure 6).

Third, the passion for e-commerce was strongly influenced by the constant desire to buy items from the online environment. This can be determined by the increasing number of online orders. It was predominantly present in all age groups because each person exhibited a passion to shop online.

To test the psychological perception of the online buyer in relation to motivational factors by econometric modeling, a conceptual model was developed and the findings have verified the hypotheses.

The data presented in Table 2 on research variables and their distribution within the age group were matrixed according to the impact classifications of the motivational factors (Table 5), resulting in the representation of the influence of the motivational factors on the minimal-maximal range (see Table 6).

**Table 6:** Means of representing the influence of motivational factors on the minimal–maximal range.

	**Perceptions of respondents under 25 years old**	**Perceptions of respondents between 25 and 55 years old**	**Perceptions of respondents having more than 55 years old**
M1-MIN	4.53	5.70	3.90
M1-Average	5.58	4.58	4.89
M1-MAX	4.35	4.67	5.57
M2-MIN	4.99	0.00	4.17
M2-Average	0.00	5.02	0.00
M2-MAX	4.29	6.33	5.75
M3-MIN	4.21	4.93	2.83
M3-Average	5.47	4.14	0.00
M3-MAX	3.27	0.00	5.78

The data were analyzed for the trend, resulting in polynomial equations of the evolutionary trend of the influence of the motivational factors. While in the 18–25 age group the trend equation had increasing values maximizing it toward the end of the interval, in the 25–55 age group, the trend showed a homogeneous behavior. However, the above 55 age group had a regressive trend, which revealed that in this age range, the sensitivity to the motivational factors of the psychological profile was maximum (Table 7).

**Table 7:** Motivational mix matrix on age scales.

	**Perceptions of respondents under 25 years old**	**Perceptions of respondents between 25 and 55 years old**	**Perceptions of respondents more than 55 years old**
M-MIN	*y* = −1.485*x*^2^ + 5.625*x* + 0.39	*y* = −1.4125*x*^2^ + 4.9625*x* + 0.655	*y* = 4.58*x*^2^ – 18.73*x* + 19.14
M-Average	*y* = 0.655*x*^2^ – 2.9683*x* + 7.8967	*y* = −1.4083*x*^2^ + 2.8983*x* + 3.98	*y* = −5.02*x*^2^ + 20.08*x* – 15.06
M-MAX	*y* = 0.2925*x*^2^ – 0.5642*x* + 4.625	*y* = 4.525*x*^2^ – 16.845*x* + 15.59	*y* = −1.3119*x*^2^ + 5.9756*x* – 0.3737

**Figure 7:**
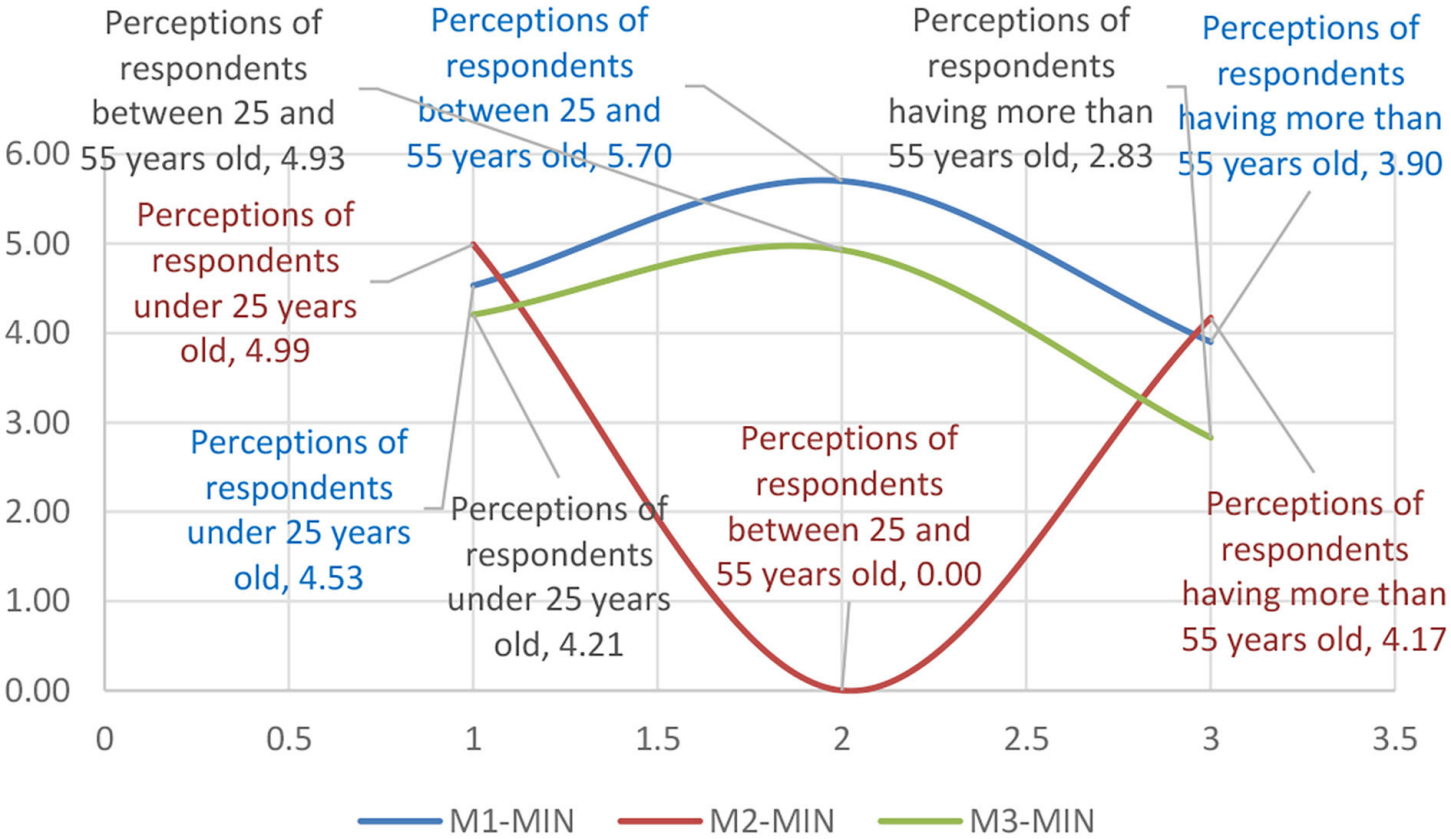
The graphical representation of the age group sensitivity to the motivational mix within the minimal range.

**Figure 8:**
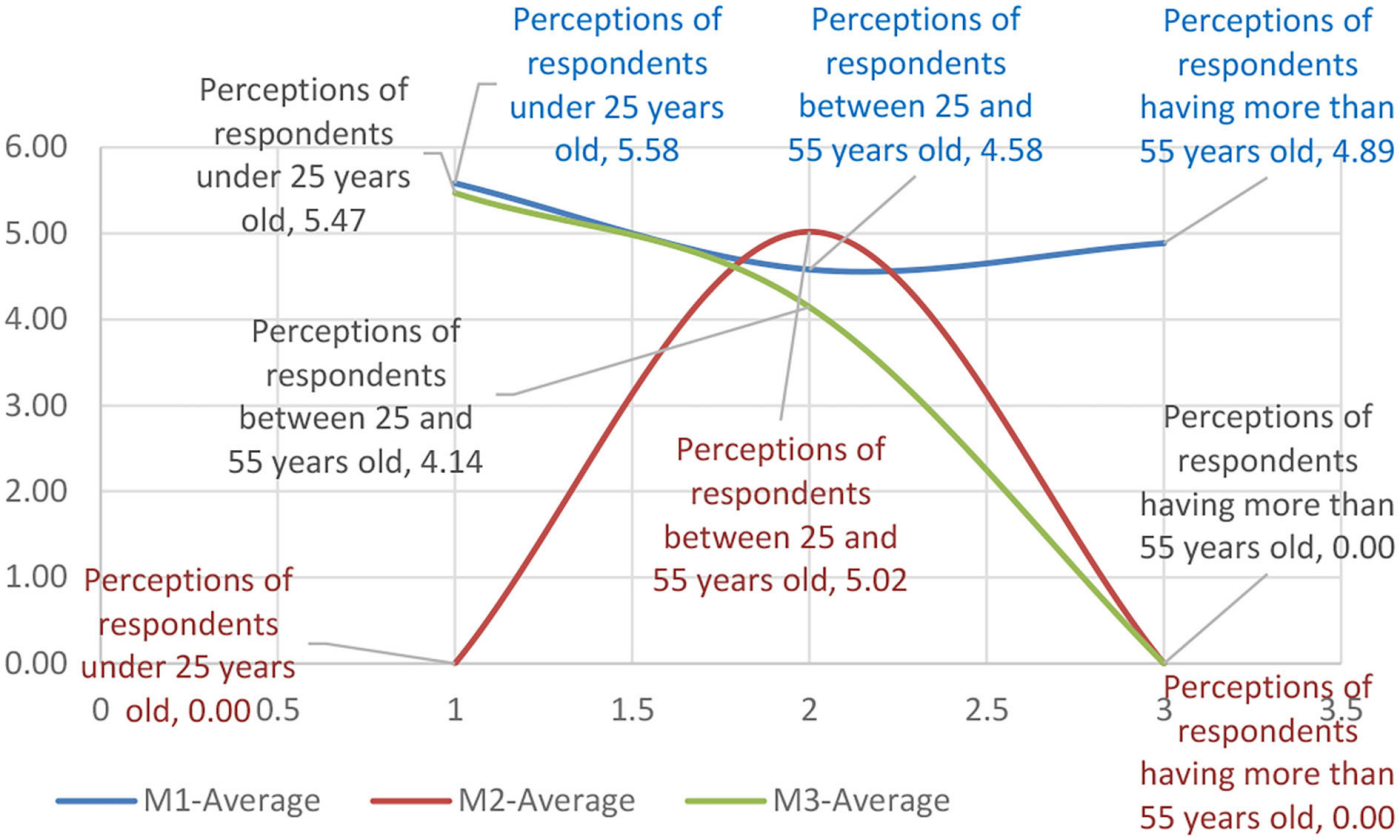
The graphical representation of the age group sensitivity to the motivational mix within the average range.

**Figure 9:**
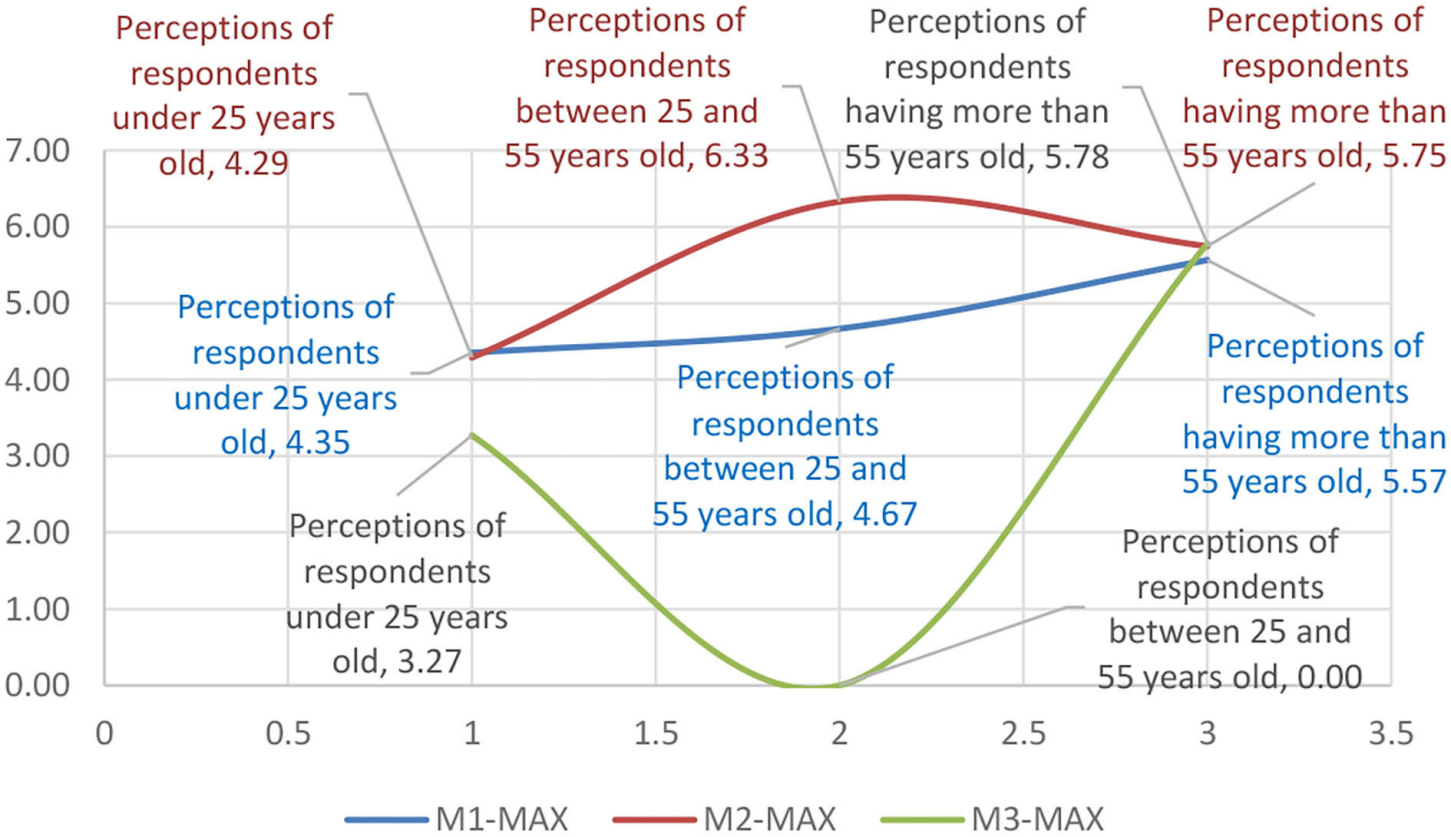
The graphical representation of the age group sensitivity to the motivational mix within the maximal range.

The data in Table 7 were further categorized by components of the motivational mix according to Figures 7–9, presented below.

From Figure 6, it can be seen that motivational composition shows significant changes depending on the age of the buyers which can be influenced by their psychological aspects (Zhao et al., 2021) and personality structure. This can change over a lifetime with accumulated experiences and knowledge (Bawack et al., 2021), as well as in relation to consumer preferences in response to managerial stimuli and the consumption process (Becker and Jaakkola, 2020).

In contrast to the minimum size, Figure 8 shows that the average distribution was significantly different on all three levels, with inverted motivational mix distribution curves. Thus, it can be observed that the behavior of the majority of the respondents fits into a more optimistic perception at the adult age level which was seen as the most homogeneous representation of the means of variation. It can be said that the sensitivity at the level of the average perception of the samples tends to respond uniquely to stimuli such as brand loyalty (Liu et al., 2021), globalization of consumer behavior (Zhang and Chang, 2021), and consumer satisfaction following purchase (Jaiswal and Singh, 2020). Other authors (Ibrahim and Wang, 2019; Li et al., 2019) presented motivational models that explain consumer preference based on personal perception or based on post-sale benefits offered by the seller.

The maximum threshold analysis showed that consumer behavior was not homogeneous and had significant differences in the margin compared to the general behavior, which is the result of the perception of purchase risks. Thus, the level of trust in the seller and the products is a risk marker (Jaiswal et al., 2018). The second marker is the buyer's interests in relation to the seller (Bi, 2019), and the third marker is the utilitarian or hedonic motivational orientation (Stein and Ramaseshan, 2020). Most respondents had a different flow state. The flow was often witnessed in the young and adult population, with the youngsters being influenced both by visual animation and by acoustics where various sound effects influenced attention toward certain products. The older adult population had a predominant flow in the course of online purchases, and this age category was strongly influenced by the media. Analyzing the average occurrence of the flow state in all age categories, an increasing trend of the flow state was observed.

The sentiments experienced in the latest online acquisition experience denoted the quasi-positive attitude of happiness, regardless of the age group of the respondents. Customer happiness, regardless of age, creates a wealth of advantages for the online seller, where the most important is their return for new shopping. This study found that the passion for e-commerce had grown, capturing all segments of the respondents' age, which was demonstrated by an increased frequency of online shopping. With advantages such as time saved, home delivery, and the possibility of returning or exchanging defective items, e-commerce, compared to other types of commerce, has reached an impressive threshold in respondents' satisfaction. Customer satisfaction is a very important factor for online commerce, based on the principle: a satisfied customer brings one, but one dissatisfied turns back ten customers (dissatisfaction would lead to a drastic drop in online shopping). This study also found that older people have a higher dose of energy than other age groups when it came to online purchasing. However, E-commerce manifests itself distinctly toward young people and adults because they are much better influenced by the promotion of articles and the joy of being served quickly and without too much effort.

Impatience is a particular trait manifested in e-commerce, while curiosity is always found in the case of online buyers. Regardless of the age group of the respondents, we noticed the customers' desire to receive the products as soon as possible and according to the anticipated image created in their minds. The research argumentation through the first *objective* of the study, namely that the online psychological profile of the online buyer influences online procurement management, is supported by the following points:

a) Online procurement management is a vector function of the standardized components of management such as the financial component, the psychological component, and the managerial-administrative component. These components coordinate between themselves within their own managerial system and react with each online purchaser. The system's stability depends on the number of interactions between the components and the matching voluntary purchase decision of the purchaser.
b) We define online procurement management as a result of voluntary and conscious acquisition actions based on the prioritized distribution of resources under favorable psychological perceptions, based on the sensory quantification of several motivational factors.

The second *objective* of the research was achieved by the quantification of the age-related motivational mix, which mathematically demonstrated that the sensitivity of the psychological profile to the motivational factors increases with age. It favorably concluded that there were differences in the psychological profile of the online buyer based on age differences and education.

For addressing the *third objective* of the research, the authors developed the psychological profiles of each age category along with the general profile, which is presented in Table 5.

## Conclusion

Consumer behavior is an area of interest for management-marketing entities because it provides critical information on the orientation of the consumer's preferences toward technological, social, qualitative, affective, and conjunctural elements of the product (Darley et al., 2010; Anaza, 2014).

In the current literature, we find approaches to consumer behavior from an affective perspective where researchers are focused on the confluence between mental processes, personality, and consumer behavior (Lemon and Verhoef, 2016; Lim et al., 2018; Molinillo et al., 2022). Our study was based on the premise that in the online environment motivational factors (affective, psychological, cognitive, and attitudinal factors) significantly influence consumer behavior and purchase decisions (Marc, 2015).

From an emotional point of view, the study demonstrates that consumers' consumption has migrated from being driven by physical needs to psychological needs. The use of social information and mass-media information exceeds the reliance on economic information by more than 500%. Consumer satisfaction has undergone a process of emotional transformation, noticeably marked by the opportunities of the online environment, and has consequently led to the adaptive modification of their purchasing patterns and behavior, which, in turn, has impacted their social state, economic dynamics, and cultural motivations. These transformations equally affect the business environment which is already facing new challenges in media advertising and promotion. Having suffered budget cuts for traditional media, and with additional allocations for increasing social media presence, more companies are opting for establishing Facebook accounts, Instagram, Twitter, etc.

Considering the costs of trading, shipping, and related online establishment costs, the prices in online commerce are actually higher than in the traditional retail segment. For instance, in the online price equation, the so-called loyalty offerings such as the moon offer, prize competitions, and bonus system play a very important role. With the main purpose of increasing the number of products sold, the price for the product being promoted is usually higher to cover the price of the bonus product, which is often not a fast-moving product. For social impact, the Black Friday and Pink Week campaigns were launched and had a huge impact in the first 2–3 years after their launch, but the psychological effect gradually diminished and buyers became increasingly reluctant to pursue the so-called promotional campaigns under the Black Friday banner. The relative value of online traffic has suffered a successive percentage decline, and the e-commerce area has even declined massively in terms of consumer interest, precisely based on the perception of Black Friday sales campaigns.

The business and commerce trend is also regressive, but the magnitude of the regression curve is much less attenuated in the absence of aggressive sales promotion, as this focuses more on the business option than on the actual sales of products, the commercial activity being consumed at the seller's premises.

Through this study, the authors contributed to the building of a psychological profile of the online buyer, a profile validated by the statistical tests applied to a representative sample of 199 persons from Romania who made online purchases on average between 6 and 10 times per year. The analyzed profiles reflect a positive attitude to online purchases, and adults aged 55 and above had a greater proclivity to shop online (21 statistical profiles of the profile vs. the general profile) while adults aged between 25 and 55 were the least inclined to shop online (3 statistics peaks of the profile vs. the general profile).

## Implications, limitations, and future directions for research

From the review of the literature, we observed that current research focused mainly on the effects of consumer behavior and less attention has been paid to the causes that determine this behavior and the actual needs of the consumer determined by various motivational stimuli of affective, psychological, cognitive, and attitudinal factors. This study, therefore, is timely due to its pragmatically oriented approach of assessing the motivational status through sensitivity analysis, which has offered significant and useful results for management-marketing decision-makers.

In the study, the research objectives and hypotheses were established. All hypotheses have been confirmed, demonstrating that the overall online buyer profile is determinable in perceptual and motivational endpoints, open to online purchases based on prior perceptions and time-based satisfaction, with a prudential nature in terms of flow states and aspects of consumer education.

The observational study showed that the management of the acquisition influences the general profile by maximizing the function of financial independence. Accordingly, the general profile is most accurately identified at the point where the financial independence is maximum (age level).

These research results can be successfully used by online marketers to create sustainable offers and to gain a competitive edge once they are launched.

Limitations of this study consist of the fact that most of the respondents have higher education (96.48%), which induces a model vulnerability in the segment of those with medium education.

As a future direction for research, we will approach the study of online consumer behavior at the European level through an extended questionnaire targeting European consumers of consumer goods and we will aim to differentiate from the sensitivity analysis the motivational status according to the regional component and according to the average household income.

## Summary statement of contribution

In this paper, we developed a statistical model for assessing consumer behavior in relation to psychological motivational factors to provide a useful tool for decision-makers responsible for online marketing strategies for products marketed in the retail system to maximize their application in marketing strategy.

The paper's importance rests in the new approach it offers to the perceptual pattern based on the age of the respondents, and assessing the impact of the motivational mix on consumers' perception through a sensitivity analysis.

## Data availability statement

The raw data supporting the conclusions of this article will be made available by the authors, without undue reservation.

## Ethics statement

Ethical review and approval was not required for the study on human participants in accordance with the local legislation and institutional requirements. Written informed consent from the participants or participants' legal guardian/next of kin was not required to participate in this study in accordance with the national legislation and the institutional requirements.

## Author contributions

VA and MZ: conceptualization and formal analysis. RI and MZ: methodology. VA and NC: validation. NC and RI: investigation and resources. VA, RI, and MZ: writing—original draft preparation. All authors have read and agreed to the published version of the manuscript.

## Conflict of interest

The authors declare that the research was conducted in the absence of any commercial or financial relationships that could be construed as a potential conflict of interest.

## Publisher's note

All claims expressed in this article are solely those of the authors and do not necessarily represent those of their affiliated organizations, or those of the publisher, the editors and the reviewers. Any product that may be evaluated in this article, or claim that may be made by its manufacturer, is not guaranteed or endorsed by the publisher.
